# Effect of low-dose dexmedetomidine on sleep quality in postoperative patients with mechanical ventilation in the intensive care unit: A pilot randomized trial

**DOI:** 10.3389/fmed.2022.931084

**Published:** 2022-08-31

**Authors:** Yue-Ming Sun, Sai-Nan Zhu, Cheng Zhang, Shuang-Ling Li, Dong-Xin Wang

**Affiliations:** ^1^Department of Critical Care Medicine, Peking University First Hospital, Beijing, China; ^2^Department of Biostatistics, Peking University First Hospital, Beijing, China; ^3^Department of Respiratory and Critical Care Medicine, Peking University First Hospital, Beijing, China; ^4^Departments of Anesthesiology and Critical Care Medicine, Peking University First Hospital, Beijing, China; ^5^Outcomes Research Consortium, Cleveland Clinic, Cleveland, OH, United States

**Keywords:** intensive care unit, ventilators, mechanical, sleep wake disorders, polysomnography, dexmedetomidine

## Abstract

**Background:**

Sleep disturbances are prevalent in patients requiring invasive mechanical ventilation in the intensive care unit (ICU) and are associated with worse outcomes. Sedative-dose dexmedetomidine may improve sleep quality in this patient population but is associated with adverse events. Herein, we tested the effect of low-dose dexmedetomidine infusion on nighttime sleep quality in postoperative ICU patients with invasive ventilation.

**Methods:**

In this pilot randomized trial, 80 adult patients who were admitted to the ICU after non-cardiac surgery and required invasive mechanical ventilation were randomized to receive either low-dose dexmedetomidine (0.1 to 0.2 μg/kg/h, *n* = 40) or placebo (*n* = 40) for up to 72 h. The primary endpoint was overall subjective sleep quality measured using the Richards–Campbell Sleep Questionnaire (score ranges from 0 to 100, with a higher score indicating better quality) in the night of surgery. Secondary outcomes included sleep structure parameters monitored with polysomnography from 9:00 PM on the day of surgery to the next 6:00 AM.

**Results:**

All 80 patients were included in the intention-to-treat analysis. The overall subjective sleep quality was median 52 (interquartile 20, 66) with placebo vs. 61 (27, 79) with dexmedetomidine, and the difference was not statistically significant (median difference 8; 95% CI: −2, 22; *P* = 0.120). Among 68 patients included in sleep structure analysis, those in the dexmedetomidine group tended to have longer total sleep time [median difference 54 min (95% CI: −4, 120); *P* = 0.061], higher sleep efficiency [median difference 10.0% (95% CI: −0.8%, 22.3%); *P* = 0.060], lower percentage of stage N1 sleep [median difference −3.9% (95% CI: −11.8%, 0.5%); *P* = 0.090], higher percentage of stage N3 sleep [median difference 0.0% (95% CI: 0.0%, 0.4%); *P* = 0.057], and lower arousal index [median difference −0.9 (95% CI −2.2, 0.1); *P* = 0.091] but not statistically significant. There were no differences between the two groups regarding the incidence of adverse events.

**Conclusion:**

Among patients admitted to the ICU after surgery with intubation and mechanical ventilation, low-dose dexmedetomidine infusion did not significantly improve the sleep quality pattern, although there were trends of improvement. Our findings support the conduct of a large randomized trial to investigate the effect of low-dose dexmedetomidine in this patient population.

**Clinical trial registration:**

ClinicalTrial.gov, identifier: NCT03335527.

## Introduction

Sleep disturbances are prevalent in patients admitted to the intensive care unit (ICU) after surgery, especially those requiring invasive mechanical ventilation (1–3). Studies using polysomnographic monitoring revealed the sleep patterns of ICU patients are characterized by a disorganized circadian rhythm, prolonged sleep latencies, fragmented sleep, decreased sleep efficiency, abnormally increased stage 1 non-rapid eye movement (N1) sleep, increased or decreased stage N2 sleep, and decreased or absent stage N3 sleep and rapid eye movement (REM) sleep (4–8). Sleep disturbances are associated with worse outcomes including increased risks of delirium and cardiac events and worsened functional recovery (9–12). Furthermore, sleep problems remain common in patients who survive critical illness. Indeed, up to 50% of ICU survivors reported sleep disturbances, which were associated with poor quality of life (13–15). However, there are no recommended medications for sleep improvement in ICU patients after surgery (16).

Dexmedetomidine is a highly selective α2-adrenergic agonist with sedative, anxiolytic, and analgesic properties. It exerts sedative effects by activating the endogenous sleep pathways and produces a state like non-rapid eye movement sleep, which is different from opioid- and benzodiazepine-induced sedation (17). In a previous study of ICU patients receiving invasive ventilation, nighttime infusion of sedative-dose dexmedetomidine (0.2–0.7 μg/kg/h) improved sleep quality by increasing sleep efficiency and N2 sleep and modifying the circadian rhythm (18). This partially explains why dexmedetomidine sedation reduces the incidence of delirium in mechanically ventilated ICU patients (19, 20). However, despite guideline recommendations (16), the use of dexmedetomidine for ICU sedation remains less common than propofol use (21). The main reason is frequent adverse events associated with its use, especially bradycardia and hypotension (20).

Side effects related to dexmedetomidine administration are dose-dependent. In our previous trial involving postoperative ICU patients without mechanical ventilation, low-dose dexmedetomidine infusion (0.1 μg/kg/h) during the night of surgery improved the sleep architecture by decreasing N1 sleep, increasing N2 sleep, prolonging total sleep time, and increasing sleep efficiency (22). In another study of postoperative ICU patients (the majority without mechanical ventilation), nighttime infusion of low-dose dexmedetomidine improved subjective sleep quality and reduced postoperative delirium (23). We hypothesized that in postoperative ICU patients requiring invasive mechanical ventilation, low-dose dexmedetomidine infusion might also improve sleep quality. The purpose of this pilot trial was to determine the effect of low-dose dexmedetomidine infusion on sleep quality in mechanically ventilated patients admitted to the ICU after non-cardiac surgery.

## Materials and methods

This was a randomized, double-blind, placebo-controlled pilot trial. The study protocol was approved by the Clinical Research Ethics Committee of Peking University First Hospital [2017(13)] and registered with ClinicalTrials.gov (NCT03335527; November 7, 2017). Written informed consent was obtained from the next of kin or legal representative of each participant. The trial was performed in the ICU of Peking University First Hospital (Beijing, China). This was a 10-bed multioccupancy ICU located at the north part of the inpatient building. There were windows in the north wall, with curtains closed in the evening. There were partition curtains between beds. Because of the large room area, the lights were usually kept on during daytime hours. The lights were turned off, and the noises weakened at night, usually from 9 PM to 6 AM next day. We had a nurse-to-patient ratio of 3:1.

### Patient recruitment

Potential participants were screened at ICU admission. We enrolled adult patients (aged 18 years or older) who were admitted with endotracheal intubation after non-cardiac surgery and had an expected duration of invasive mechanical ventilation for at least 12 h (admitted before 9:00 PM on the day of surgery and extubated after 6:00 AM on the 1st day after surgery). Patients who met any of the following criteria were excluded: refused to participate; pregnant; a preoperative history of schizophrenia, epilepsy, parkinsonism, or myasthenia gravis; inability to communicate (coma, profound dementia, or language barrier); brain injury or neurosurgery; left ventricular ejection fraction <30%, sick sinus syndrome, severe sinus bradycardia (<50 beats per min), or second-degree or higher atrioventricular block without pacemaker; systolic blood pressure <90 mmHg in spite of continuous infusion of vasopressors; serious hepatic dysfunction (Child–Pugh class C), serious renal dysfunction (undergoing dialysis before surgery), or less likely to survive for >24 h; sleep disorders (requirement of hypnotics/sedatives during the last month) or a history of obstructive sleep apnea syndrome; or other conditions that were considered unsuitable for study participation.

### Randomization, study drug administration, and perioperative care

An independent biostatistician generated random numbers in a 1:1 ratio with a block size of four using SAS 9.4 software (SAS Institute, Cary, NC). Study drugs (dexmedetomidine hydrochloride 200 μg/2 ml and normal saline 2 ml) were provided as clear aqueous solutions in the same 3-ml vials (manufactured by Jiangsu Hengrui Medicine Co, Ltd, Jiangsu, China). The study drugs were sequentially numbered according to the randomization results by a pharmacist who otherwise did not participate in the study. The blinding codes were sealed in sequentially numbered opaque envelopes (the same number as that of the corresponding study drug) and stored by a study coordinator until the end of the trial.

During the study period, the study coordinator distributed the study drugs according to the sequence of patient recruitment, and in this way, the consecutively enrolled participants were randomly assigned to receive either dexmedetomidine or placebo. The study drugs were diluted with normal saline to 50 ml and administered as continuous intravenous infusions at an initial rate of 0.025 ml/kg/h (0.1 μg/kg/h for dexmedetomidine). The infusion rate was increased to 0.05 ml/kg/h (0.2 μg/kg/h for dexmedetomidine) 30 min later if no hypotension (SBP <90 mmHg or a decrease >30% from baseline) or bradycardia (heart rate <50 beats per min) occurred. Study drug infusion was initiated from trial recruitment on the day of ICU admission, continued during invasive mechanical ventilation, and the spontaneous breathing test, for a period of up to 3 days. All investigators, healthcare team members, and patients were masked from study group assignment.

As a routine practice, postoperative analgesia was provided with a patient-controlled intravenous or epidural analgesia pump. Supplemental morphine was administered as bolus injection (2.5–10 mg) when necessary. Non-steroid anti-inflammatory drugs were administered for patients without contraindications. The target was to maintain the numeric rating scale (an 11-point scale, where 0 = no pain and 10 = the worst pain) ≤ 4 or the behavior pain scale ≤ 6. The behavior pain scale includes three subscales, namely, facial expression, upper limb movement, and compliance with ventilation. The score of each subscale ranges from 1 to 4. The total score ranges from 3 to 12, with a higher score indicating a more severe pain-related experience (24). After achieving adequate analgesia, patients still requiring sedation were provided with propofol infusion at a rate of 0.3–4 mg/kg/h; the target was to maintain Richmond Agitation-Sedation Scale [RASS; score ranges from −5 (unarousable) to +4 (combative), and 0 indicates alert and calm] (25) between −2 and +1 (assessed hourly by trained nurses). As a routine practice, we had a daytime rest from 12 AM to 13 PM, although not necessarily for every patient; nighttime nursing care was clustered, when possible, in order to decrease sleep interference. The sedative infusion was stopped each morning in order to conduct the spontaneous breathing test for early extubation. Other managements were provided as per routine.

In case of an emergency (e.g., unexpected, rapid deterioration of the patient's clinical condition), the attending intensivists could decrease or terminate study drug infusion and/or request to unmask blinding. In such case, the reasons were recorded in the case report forms, but patients were included in the final analyses.

### Baseline and perioperative data

Baseline data included sociodemographic parameters, surgical diagnoses, preoperative comorbidities, and main laboratory test results. The severity of comorbid diseases and general status were evaluated using the Charlson Comorbidity Index (26), New York Heart Association (NYHA) functional classification, and American Society of Anesthesiologists (ASA) physical status classification.

Intraoperative data included the type and duration of anesthesia, medications administered during anesthesia, estimated blood loss, transfusion of blood products, and location and duration of surgery. Operative stress was stratified into five categories according to the Operative Stress Score, that is, very low stress, low stress, moderate stress, high stress, and very high stress (27). Postoperative data included the Acute Physiology and Chronic Health Evaluation (APACHE) II score at ICU admission, use of patient-controlled analgesia after surgery, use of other analgesics and sedatives within 7 days, and the duration of study drug administration.

### Polysomnographic monitoring

Polysomnographic monitoring was performed from 9:00 PM to 6:00 AM during the night of surgery with an EEG/PSG Recording System (SOMNO screen plus, SOMNO medics GmbH, Randersacker, Germany). Electrodes were attached by qualified investigators. The polysomnographic monitoring included eight-channel electroencephalogram (C3, C4, F3, F4, O1, O2, M1, and M2), two-channel electrooculogram (E1 and E2), and three-channel chin electromyogram (Chin1, Chin2, and Chin3). The monitored data were automatically recorded and processed according to the American Academy of Sleep Medicine (AASM) manual (28). A qualified sleep physician who was blinded to study group assignment and did not participate in perioperative management was responsible to identify sleep stages according to the manual.

The whole monitored period was divided into wakefulness, non-rapid eye movement sleep, and rapid eye movement sleep. Non-rapid eye movement sleep was further divided into three stages, namely, N1, N2, and N3. The total sleep time was defined as the summary of time spent in any sleep stage during the monitoring period. Sleep efficiency was calculated as the summary of each sleep stage divided by total sleep monitoring time. The percentages of each sleep stage were calculated as the durations of each sleep stage divided by the total sleep time. The arousal index was calculated as arousal times divided by total sleep time.

### Outcome assessments

The primary outcome was the overall subjective sleep quality during the night of surgery, which was assessed using the Richards–Campbell Sleep Questionnaire (RCSQ) in the first postoperative morning between 6:00 AM and 10:00 AM (29, 30). The RCSQ is a self-reported measure that evaluated perception of nighttime sleep in five items, namely, sleep depth, sleep latency, number of awakenings, returning to sleep, and overall sleep quality. Each item was assessed with a 100-mm visual analog scale (VAS; score ranges from 0 to 100, with higher scores representing better sleep). The mean score of the five items represents the overall RCSQ score. The RCSQ also included a sixth item, not included in the overall score, that evaluated perceived nighttime noise (score ranges from 0 to 100, where 0 = “very noisy” and 100 = “very quiet”). For patients who were not extubated the next morning, self-evaluation was performed after stopping sedation and regained consciousness. The usefulness of RCSQ in assessing sleep quality of ICU patients with or without invasive mechanical ventilation has been validated in previous studies (29, 30). It is concluded that RCSQ is a reliable alternative to polysomnography in this patient population (31). According to results of a previous study, a 10-mm change in the 100-mm visual analog scale of the sleep quality is considered clinically important (32).

Secondary outcomes included daily results of individual RCSQ items during postoperative days 0 to 7, the duration of mechanical ventilation and ICU stay after surgery, the incidence of delirium within the first 7 postoperative days, the length of stay in hospital after surgery, the incidence of non-delirium complications within 30 days, and all-cause 30-day mortality. Sleep quality was assessed daily in the morning between 6:00 AM and 10:00 AM. Delirium was assessed two times daily (6:00 AM to 10:00 AM and 6:00 PM to 8:00 PM) using the Confusion Assessment Method for the ICU (CAM-ICU) (23, 33, 34). Before assessing delirium, sedation or agitation was assessed using RASS. Deeply sedated or unarousable patients (RASS −4 or −5) were recorded as comatose and not assessed for delirium. Non-delirium complications were defined as new-onset medical conditions other than delirium that were deemed harmful and required therapeutic intervention, that is, grade II or higher on the Clavien–Dindo classification (35).

Apart from sleep structure parameters, other predefined outcomes included quality of life, cognitive function, and quality of sleep in 30-day survivors. Quality of life was assessed with the World Health Organization Quality of Life-brief version (WHOQOL-BREF; a 24-item questionnaire that provides assessments of the quality of life in physical, psychological, and social relationship and environmental domains. For each domain, the score ranges from 0 to 100, with a higher score indicating better function) (36). Cognitive function was assessed using the Telephone Interview for Cognitive Status-modified (TICS-m; a 12-item questionnaire that verbally assesses global cognitive function *via* telephone. The score ranges from 0 to 50, with higher score indicating better function) (37). Sleep quality was assessed using the Pittsburgh Sleep Quality Index (PSQI; a seven-item questionnaire consisting of 19 self-rated questions that assesses sleep quality over the last month, each weighted equally on a 0–3 scale; higher scores indicate worse sleep quality) (38).

Adverse events were monitored during study drug infusion. Specifically, we evaluated bradycardia (heart rate <50 beats per minute), hypotension (systolic blood pressure <90 mmHg or a decrease of more than 30% from baseline), tachycardia (heart rate >120 beats per minute), hypertension (systolic blood pressure >180 mmHg or an increase of more than 30% from baseline), respiratory depression (respiratory rate <10 breaths per minute), desaturation (pulse oxygen saturation <90%), oversedation (a RASS score ≤ -3), and others. Severe adverse events indicate those that lead to death, threat of life, persistent disability or dysfunction, prolonged hospital stay, or other severe events.

### Statistical analysis

#### Sample size estimation

According to our previous study, low-dose dexmedetomidine infusion improved the score of subjective sleep quality (assessed with the numeric rating scale, an 11-point scale, where 0 indicated the best sleep and 10 indicated the worst sleep) from 4.32 ± 2.55 in the placebo group to 2.47 ± 2.42 in the dexmedetomidine group in patients admitted to the ICU after surgery (23). We presumed an effect size of 18.5 points on a 0- to 100-point scale in the present trial. With the significance level set at 0.05 (two-sided) and power at 0.80, the calculated sample size was 30 patients in each group (1:1 ratio). Considering a dropout rate of about 25%, we planned to enroll 40 patients in each group. This pilot trial was conducted to guide a future large randomized trial.

#### Outcome analysis

Continuous data were evaluated for normality using the Shapiro–Wilk test and are presented as mean ± SD or median (interquartile range). Categorical variables are presented as *n* (%). Outcome analysis was performed in the intent-to-treat population, that is, all patients were analyzed in the group to which they were randomized. For the primary outcome, analysis was also performed in the per-protocol population, in which case patients with protocol deviation were excluded. For sleep structure results, analysis was performed in patients who completed polysomnographic monitoring.

The primary endpoint (overall RCSQ score) was compared using the Mann–Whitney *U*-test. The median difference between groups and 95% CI of the difference were calculated with Hodges–Lehmann estimators. For other outcomes, continuous variables were analyzed with independent samples *t*-test or Mann–Whitney *U*-test. Categorical variables were analyzed with chi-squared analysis, continuity correction chi-squared test, or Fisher's exact test. Time-to-event outcomes were analyzed with Kaplan–Meier survival analyses, with differences between groups assessed by using the log-rank test. For results of individual RCSQ items from postoperative days 1 to 7, repeated-measures analysis of variance was used to analyze the effect of group × time. All tests were two-sided. *P*-values of <0.05 were considered statistically significant. Statistical analysis was performed with the SPSS 25.0 software package (Inc, Chicago, IL).

## Results

**Figure 1:**
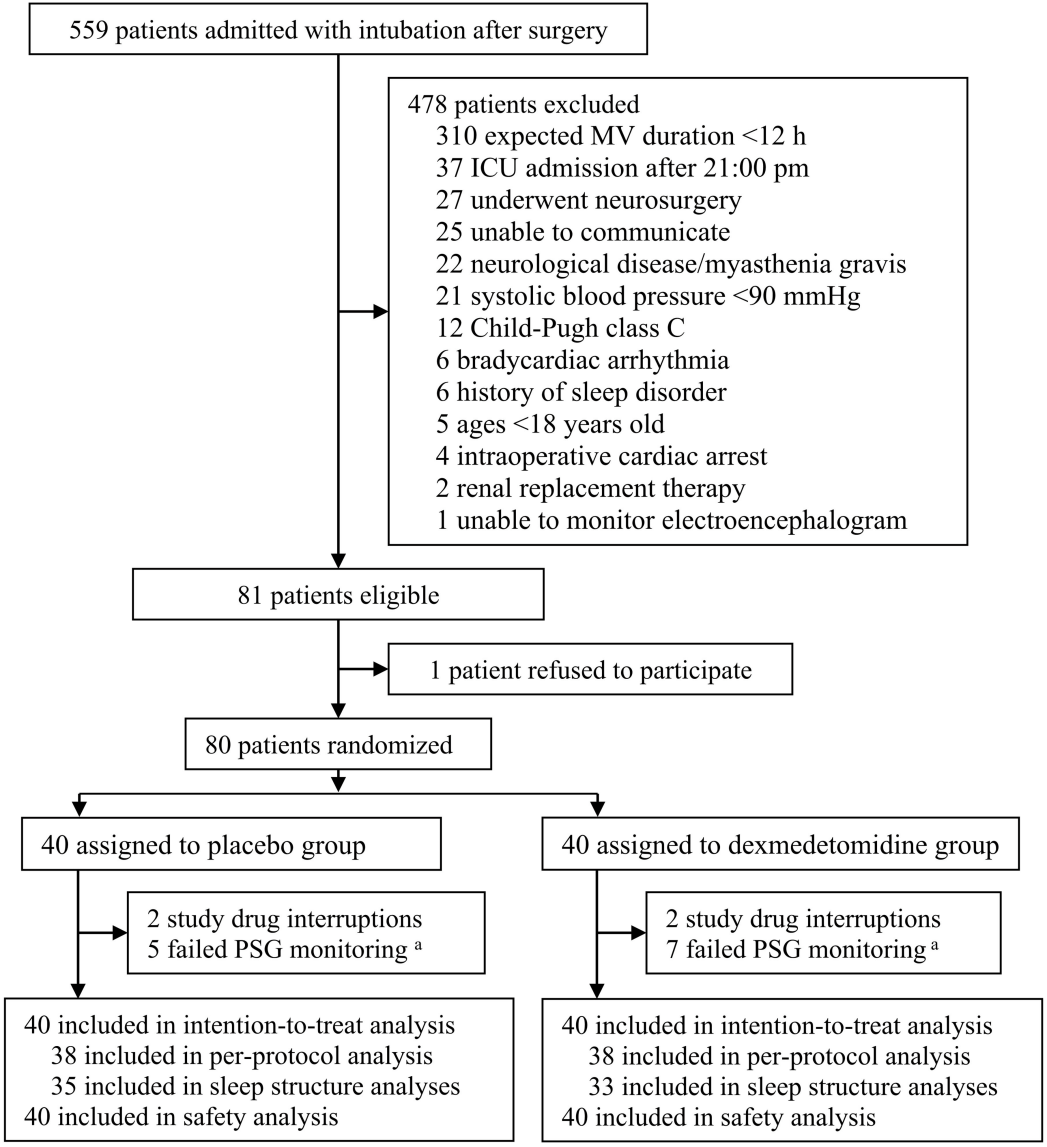
Flowchart of the trial. MV, mechanical ventilation; ICU, intensive care unit; PSG, polysomnography. ^a^Due to electrode detachment or signal interference.

From 17 November 2017 to 14 April 2019, 559 patients who were admitted to the ICU with endotracheal intubation after surgery and required mechanical ventilation were screened. Of these, 80 patients were enrolled and randomized to receive either placebo (*N* = 40) or dexmedetomidine (*N* = 40). All these patients were included in the intention-to-treat and safety analysis. During the study period, four patients with premature drug interruption were excluded from the per-protocol analysis; 12 patients had failed polysomnographic monitoring and were excluded from sleep structure analysis. All patients completed subjective sleep quality assessment in the morning. During the follow-up period, three patients died within 30 days, and one patient refused the 30-day follow-up test. The last patient follow-up was performed on 14 May 2019 (Figure 1).

The two groups were well-balanced regarding baseline and perioperative variables in all enrolled patients and patients included in sleep structure analysis, except that the duration of study drug infusion was shorter in the dexmedetomidine group in those included in sleep structure analysis (Tables 1, 2). The mean infusion rate of dexmedetomidine (in the dexmedetomidine group) was 0.2 μg/kg/h of all enrolled patients and of those included in the sleep structure analysis.

**Table 1:** Baseline characteristics.

	**All enrolled patients**			**Patients for sleep structure analysis**		
	**Placebo group** **(*N* = 40)**	**Dexmedetomidine group** **(*N* = 40)**	***P*-value**	**Placebo group** **(*N* = 35)**	**Dexmedetomidine group** **(*N* = 33)**	***P*-value**
Age (y)	65.9 ± 12.2	70.6 ± 15.6	0.143	65.9 ± 12.6	69.1 ± 16.6	0.380
Male sex	21 (52.5%)	20 (50.0%)	0.823	18 (51.4%)	17 (51.5%)	0.994
Body mass index (kg/m^2^)	23.5 ± 4.7	23.9 ± 3.8	0.613	23.1 ± 4.7	23.8 ± 4.1	0.540
Han nationality	36 (90.0%)	36 (90.0%)	0.327	33 (94.3%)	29 (87.9%)	0.421
Education (y)	10.6 ± 4.2	10.3 ± 4.3	0.792	10.3 ± 4.3	9.9 ± 4.4	0.701
Preoperative comorbidity						
Stroke	6 (15.0%)	11 (27.5%)	0.172	6 (17.1%)	9 (27.3%)	0.314
Chronic obstructive pulmonary diseases	5 (12.5%)	4 (10.0%)	>0.999	5 (14.3%)	3 (9.1%)	0.710
Asthma	1 (2.5%)	3 (7.5%)	0.608	1 (2.9%)	3 (9.1%)	0.349
Hypertension	21 (52.5%)	22 (55.0%)	0.823	18 (51.4%)	17 (51.5%)	0.994
Coronary heart disease	8 (20.0%)	11 (27.5%)	0.431	7 (20.0%)	7 (21.2%)	0.902
Arrhythmia	2 (5.0%)	5 (12.5%)	0.429	2 (5.7%)	3 (9.1%)	0.668
Diabetes	13 (32.5%)	13 (32.5%)	>0.999	10 (28.6%)	10 (30.3%)	0.876
Abnormal renal function^a^	0 (0.0%)	3 (7.5%)	0.239	0 (0.0%)	3 (9.1%)	0.109
Chronic smoking^b^	18 (45.0%)	10 (25.0%)	0.061	16 (45.7%)	9 (27.3%)	0.115
Alcoholism^c^	12 (30.0%)	6 (15.0%)	0.108	10 (28.6%)	6 (18.2%)	0.313
History of surgery	29 (72.5%)	30 (75.0%)	0.799	25 (71.4%)	23 (69.7%)	0.876
Surgical diagnosis			0.986			0.846
Gastrointestinal cancer^d^	14 (35.0%)	14 (35.0%)		11 (31.4%)	12 (36.4%)	
Urogenital cancer^e^	10 (25.0%)	9 (22.5%)		8 (22.9%)	5 (15.2%)	
Other cancer or sarcoma^f^	5 (12.5%)	6 (15.0%)		5 (14.3%)	6 (18.2%)	
Non-cancer diseases^g^	11 (27.5%)	11 (27.5%)		11 (31.4%)	10 (30.2%)	
Charlson Comorbidity index, score^h^	1 (0, 2)	2 (0, 3)	0.349	1 (0, 3)	2 (0, 4)	0.349
NYHA classification			0.944			0.915
I	14 (35.0%)	14 (35.0%)		14 (40.0%)	14 (42.4%)	
II	21 (52.5%)	20 (50.0%)		17 (48.6%)	15 (45.5%)	
III	5 (12.5%)	6 (15.0%)		4 (11.4%)	4 (12.1%)	
ASA classification			0.852			0.939
II	16 (40.0%)	13 (32.5%)		14 (40.0%)	12 (36.4%)	
III	20 (50.0%)	25 (62.5%)		18 (51.4%)	19 (57.6%)	
IV	4 (10.0%)	2 (5.0%)		3 (8.6%)	2 (6.0%)	
Laboratory test results						
Hematocrit, %	36.6 ± 7.6	36.7 ± 7.0	0.960	36.2 ± 7.3	37.0 ± 7.5	0.683
Albumin, g/L	38.0 ± 6.4	37.7 ± 6.7	0.831	38.3 ± 5.8	37.6 ± 6.6	0.658
Glucose <4.0 or >10.0 mmol/L	4 (10.8%) [3]	6 (15.4%) [1]	0.802	4 (12.1%) [2]	4 (12.5%) [1]	>0.999
Na^+^ <135.0 or >145.0 mmol/L	8 (20.5%) [1]	5 (12.5%)	0.337	6 (17.6%) [1]	3 (9.1%)	0.476
K^+^ <3.5 or >5.5 mmol/L	6 (15.4%) [1]	4 (10.0%)	0.703	5 (14.7%) [1]	4 (12.1%)	>0.999
Creatinine >133 μmol/L	2 (5.1%)	4 (10.0%)	0.675	2 (5.9%)	3 (9.1%)	0.668

Data are mean ± SD, median (interquartile range), or n (%). Numbers in square brackets indicate patients with missing data.

NYHA, New York Heart Association; ASA, American Society of Anesthesiologists.

a Serum creatinine >177 μmol/L.

b Smoking half a pack of cigarettes per day for at least 1 year, including current or past smokers.

c Drinking 100 ml alcohol per day for at least 1 year, including current or past drinkers.

d Included esophageal cancer, gastric cancer, colonic cancer, and rectal cancer.

e Include renal cancer, ureteric cancer, bladder cancer, prostatic cancer, and ovarian cancer.

f Include lung carcinoma, malignant pheochromocytoma, plasma cell myeloma, leiomyosarcoma, cutaneous carcinoma, osteosarcoma, cholangiocarcinoma, and pancreatic head carcinoma.

g ncluded uterine fibroids, pyloric obstruction, renal calculus, pheochromocytoma, intestinal obstruction, gall stone, gastrointestinal stromal tumors, scoliosis, appendicular adenoma, ganglioneuroma, lumbar intervertebral disc, adrenocortical adenoma, spondylitis, bunamiodyl, and neck infection.

h Calculated according to the updated Charlson Comorbidity Index (26).

**Table 2:** Perioperative variables.

	**All enrolled patients**			**Patients for sleep structure analysis**		
	**Placebo group** **(*N* = 40)**	**Dexmedetomidine group** **(*N* = 40)**	***P*-value**	**Placebo group** **(*N* = 35)**	**Dexmedetomidine group** **(*N* = 33)**	***P*-value**
Type of anesthesia			0.565			0.742
General alone	26 (65.0%)	23 (57.5%)		21 (60.0%)	18 (54.5%)	
Combined epidural-general	0 (0.0%)	1 (2.5%)		0 (0.0%)	1 (3.0%)	
Combined peripheral-general	14 (35.0%)	16 (40.0%)		14 (40.0%)	14 (42.5%)	
Duration of anesthesia (h)	5.2 ± 2.0	5.2 ± 2.2	0.985	5.3 ± 2.0	5.4 ± 2.3	0.869
Medication during anesthesia						
Use of sevoflurane	22 (55.0%)	22 (55.0%)	>0.999	20 (57.1%)	17 (51.5%)	0.641
Use of N_2_O	25 (62.5%)	22 (55.0%)	0.496	22 (62.9%)	19 (57.6%)	0.656
Use of propofol	40 (100.0%)	40 (100.0%)	–	35 (100.0%)	33 (100.0%)	–
Dose of propofol (mg)	800 (573, 960)	764 (481, 1,187)	0.733	775 (565, 933)	776 (516, 1,270)	0.787
Use of etomidate	31 (77.5%)	30 (75.0%)	0.793	27 (77.1%)	25 (75.8%)	0.893
Dose of etomidate (mg)^a^	12 (10, 15)	10 (8, 16)	0.753	12 (10, 16)	10 (8, 15)	0.388
Use of sufentanil	36 (90.0%)	39 (97.5%)	0.356	31 (88.6%)	32 (97.0%)	0.357
Dose of sufentanil (μg)^a^	35 (22, 51)	35 (25, 45)	0.588	35 (21, 52)	35 (25, 45)	0.694
Use of remifentanil	23 (57.5%)	29 (72.5%)	0.160	21 (60.0%)	24 (72.7%)	0.210
Dose of remifentanil (μg)^a^	180 (59, 334)	215 (117, 482)	0.428	200 (56, 340)	233 (127, 545)	0.301
Use of flurbiprofen axetil	18 (45.0%)	19 (47.5%)	0.823	15 (42.9%)	17 (51.5%)	0.475
Dose of flurbiprofen axetil (mg)^a^	50 (50, 63)	50 (50, 50)	0.391	50 (50, 100)	50 (50, 50)	0.112
Estimated blood loss, ml	100 (0, 575)	100 (50, 725)	0.555	100 (0, 500)	100 (35, 475)	0.663
Intraoperative blood transfusion	13 (32.5%)	14 (35.0%)	0.813	11 (31.4%)	12 (36.4%)	0.667
Duration of surgery (h)	3.8 ± 1.9	3.9 ± 2.1	0.811	3.9 ± 2.0	4.1 ± 2.2	0.681
Location of surgery			0.639			0.355
Intrathoracic	3 (7.5%)	3 (7.5%)		3 (8.6%)	3 (9.1%)	
Intraabdominal	27 (67.5%)	26 (65.0%)		25 (71.4%)	21 (63.6%)	
Pelvic	6 (15.0%)	4 (10.0%)		4 (11.4%)	4 (12.1%)	
Spinal and extremital	4 (10.0%)	5 (12.5%)		3 (8.6%)	3 (9.1%)	
Superficial	0 (0.0%)	2 (5.0%)		0 (0.0%)	2 (6.1%)	
Level of operative stress^b^			0.543			0.906
Very low stress	0 (0.0%)	0 (0.0%)		0 (0.0%)	0 (0.0%)	
Low stress	1 (2.5%)	2 (5.0%)		1 (2.9%)	1 (3.0%)	
Moderate stress	17 (42.5%)	16 (40.0%)		15 (42.9%)	13 (39.4%)	
High stress	16 (40.0%)	19 (47.5%)		14 (40.0%)	16 (48.5%)	
Very high stress	6 (15.0%)	3 (7.5%)		5 (14.2%)	3 (9.1%)	
APACHE II score on ICU admission	11 ± 4	12 ± 4	0.718	11 ± 4	11 ± 4	0.874
Patient-controlled analgesia			0.235			0.097
None	8 (20.0%)	14 (35.0%)		6 (17.1%)	13 (39.4%)	
Intravenous	32 (80.0%)	25 (62.5%)		29 (82.9%)	19 (57.6%)	
Epidural	0 (0.0%)	1 (2.5%)		0 (0.0%)	1 (3.0%)	
Other analgesics within 7 days						
Flurbiprofen axetil	18 (45.0%)	16 (40.0%)	0.651	16 (45.7%)	13 (39.4%)	0.598
Parecoxib	7 (17.5%)	10 (25.0%)	0.412	7 (20.0%)	9 (27.3%)	0.480
Morphine	7 (17.5%)	6 (15.0%)	0.762	7 (20.0%)	5 (15.2%)	0.600
Others^c^	6 (15.0%)	11 (27.5%)	0.172	5 (14.3%)	9 (27.3%)	0.186
Other sedatives within 7 days						
Propofol	7 (17.5%)	3 (7.5%)	0.176	6 (17.1%)	3 (9.1%)	0.478
Benzodiazepines	1 (2.5%)	1 (2.5%)	1.000	1 (2.9%)	1 (3.0%)	>0.999
Duration of study drug infusion (h)	12 ± 3	10 ± 4	0.065	12 ± 2	10 ± 4	0.010
Mean dexmedetomidine rate (μg/kg/h)	–	0.16 ± 0.06	–	–	0.15 ± 0.06	–
Total sleep monitoring time (min)	–	–	–	540 ± 1	540 ± 1	0.393

Data are mean ± SD, median (interquartile range), or n (%). P-value in bold indicates <0.05.

ICU, intensive care unit; APACHE, acute physiology and chronic health evaluation.

a Results of users.

b Stratified into five categories according to the Operative Stress Score, that is, very low stress, low stress, moderate stress, high stress, and very high stress.

c Included remifentanil, bucinnazine, pethidine, codeine, tramadol, and oxycodone.

The overall RCSQ score in the night of surgery was median 52 (interquartile range: 20, 66) with placebo vs. 61 (27, 79) with dexmedetomidine; patients given dexmedetomidine had a slightly higher overall RCSQ score, although the difference was not statistically significant [median difference 8 (95% CI: −2, 22), *P* = 0.120]. Per-protocol analysis also showed that patients with dexmedetomidine had a slightly higher overall RCSQ score but not statistically significant: 54 (21, 66) with placebo vs. 61 (28, 78) with dexmedetomidine; median difference 7 (95% CI: −3, 22); *P* = 0.175. Regarding the individual RCSQ items in the night of surgery, the scores of awakenings [median difference 12 (95% CI: 0, 27); *P* = 0.052] and overall sleep quality [median difference 9 (95% CI: −2, 28); *P* = 0.099] were slightly better in the dexmedetomidine group, but not statistically significant (Table 3). Daily results of individual RCSQ items showed no significant differences between the two groups (Supplementary Table).

**Table 3:** Outcome analyses.

	**Placebo group** **(*N* = 40)**	**Dexmedetomidine group** **(*N* = 40)**	**Estimated difference** **(95% CI)**	***P*-value**
**Primary outcome**				
Overall RCSQ score^a^	52 (20, 66)	61 (27, 79)	Median D = 8 (−2, 22)	0.120
Overall RCSQ score^a^ (per-protocol analysis)	54 (21, 66) (*N* = 38)	61 (28, 78) (*N* = 38)	Median D = 7 (−3, 22)	0.175
**Secondary outcomes**				
RCSQ items^a^				
Sleep depth	16 (8, 80)	15 (10, 85)	Median D = 1 (−4, 8)	0.769
Sleep latency	73 (10, 83)	80 (12, 86)	Median D = 3 (−3, 10)	0.281
Awakenings	36 (10, 66)	65 (22, 82)	Median D = 12 (0, 27)	0.052
Returning to sleep	77 (11, 84)	80 (16, 87)	Median D = 3 (−3, 11)	0.324
Overall sleep quality	49 (10, 80)	70 (22, 86)	Median D = 9 (−2, 28)	0.099
Noise	81 (55, 87)	85 (80, 88)	Median D = 4 (−1, 10)	0.093
Duration of MV after surgery (h)	13 (12, 14)	13 (12, 13)	HR = 0.99 (0.97, 1.01)	0.648
Extubation before next 8 AM	28 (70.0%)	26 (65.0%)	HR = 0.86 (0.46, 1.62)	0.633
Length of stay in ICU after surgery (h)	48 (36, 72)	48 (24, 60)	HR = 0.97 (0.88, 1.06)	0.443
Delirium within 7 days	5 (12.5%)	6 (15.0%)	RR = 1.24 (0.34, 4.43)	0.745
Length of stay in hospital after surgery (d)	10 (9, 17)	11 (9, 14)	HR = 0.98 (0.94, 1.01)	0.179
Non-delirium complications within 30 days	10 (25.0%)	7 (17.5%)	RR = 0.64 (0.22, 1.88)	0.412
All-cause 30-d mortality	2 (5.0%)	1 (2.5%)	RR = 0.49 (0.04, 5.60)	>0.999
**Other predefined outcomes**	(*N* = 37)	(*N* = 39)		
Quality of life in 30-day survivors ^b^				
Physical domain	59 ± 20	53 ± 18	Mean D = −7 (−15, 2)	0.133
Psychological domain	65 ± 14	63 ± 13	Mean D = −2 (−8, 4)	0.476
Social relationships domain	57 ± 9	58 ± 12	Mean D = 1 (−4, 6)	0.654
Environment domain	63 ± 14	62 ± 13	Mean D = −1 (−7, 5)	0.781
Cognitive function at 30 days^c^	29 ± 9	30 ± 9	Mean D = 1 (−3, 5)	0.546
Pittsburgh Sleep Quality Index at 30 days^d^	7 (4, 11)	8 (5, 12)	Median D = 1 (−1, 4)	0.231

Data are median (interquartile range), n (%), or mean ± SD.

RCSQ, Richards–Campbell Sleep Questionnaire; D, difference; HR, hazard ratio; RR, relative risk; MV, mechanical ventilation; ICU, intensive care unit.

a Subjective sleep quality was assessed with the RCSQ in the night of surgery. The RCSQ is a five-item questionnaire. Responses are recorded on a 100-mm visual analog scale, with higher scores representing better sleep. The mean of these five items represents the overall RCSQ score. The RCSQ also included a sixth item, not included in the overall score, evaluating perceived nighttime noise (visual analog scale range: 0 for “very noisy” to 100 for “very quiet”).

b Assessed with the World Health Organization Quality of Life-brief version, a 24-item questionnaire that provides assessments of the quality of life in physical, psychological, and social relationship, and environmental domains. For each domain, the score ranges from 0 to 100, with a higher score indicating better function.

c Assessed with the Telephone Interview for Cognitive Status-modified, a 12-item questionnaire that provides an assessment of global cognitive function by verbal communication via telephone; scores range from 0 to 50, with a higher score indicating better function.

d Pittsburgh Sleep Quality Index included seven items, each weighted equally on a 0–3 scale, resulting a total score from 0 to 21; higher scores indicate worse sleep quality.

There were no significant differences between the two groups regarding other secondary outcomes, including the duration of mechanical ventilation, length of stay in the ICU and hospital, incidence of delirium within 7 days, length of stay in hospital after surgery, incidence of non-delirium complications within 30 days, and all-cause 30-day mortality. At 30 days after surgery, the quality of life as assessed with the WHOQOL-BREF, the cognitive function as assessed with the TICS-m, and the sleep quality over 30 days as assessed with the Pittsburgh Sleep Quality Index did not differ between the two groups (Table 3).

Among patients included in the sleep architecture analyses, those in the dexmedetomidine group tended to have longer total sleep time [median difference 54 min (95% CI: −4, 120); *P* = 0.061], higher sleep efficiency [median difference 10.0% (95% CI: −0.8%, 22.3%); *P* = 0.060], lower percentage of stage N1 sleep [median difference −3.9% (95% CI: −11.8%, 0.5%); *P* = 0.090], higher percentage of stage N3 sleep [median difference 0.0% (95% CI: 0.0%, 0.4%); *P* = 0.057], and lower arousal index [median difference −0.9 (95% CI: −2.2, 0.1); *P* = 0.091], but the differences were not statistically significant (Table 4).

**Table 4:** Sleep architecture analyses.

	**Placebo group** **(*N* = 35)**	**Dexmedetomidine group** **(*N* = 33)**	**Estimated difference** **(95% CI)**	***P*-value**
Total sleep time (min)^a^	184 (124, 313)	261 (158, 347)	Median D = 54 (−4, 120)	0.061
Sleep efficiency (%)^b^	34.0 (22.9, 57.9)	48.3 (29.2, 64.2)	Median D = 10 (−0.8, 22.3)	0.060
Duration of stage N1 sleep (min)	14 (3, 38)	11 (3, 29)	Median D = −2 (−11, 4)	0.484
Percentage of stage N1 sleep (%)^c^	12.1 (1.5, 26.8)	5.6 (1.1, 11.7)	Median D = −3.9 (−11.8, 0.5)	0.090
Duration of stage N2 sleep (min)	152 (82, 264)	237 (102, 307)	Median D = 43 (−13, 107)	0.143
Percentage of stage N2 sleep (%)^d^	84.5 (68.1, 95.8)	89.9 (71.1, 95.3)	Median D = 1 (−5.2, 10.0)	0.659
Duration of stage N3 sleep (min)	0 (0, 0)	0 (0, 16)	Median D = 0 (0, 0)	0.054
Percentage of stage N3 sleep (%)^e^	0.0 (0.0, 0.0)	0.0 (0.0, 7.1)	Median D = 0.0 (0.0, 0.4)	0.057
Presence of stage N3 sleep	7 (20.0%)	13 (36.1%)	RR = 1.32 (0.96, 1.82)	0.079
Duration of stage REM sleep (min)	0 (0, 0)	0 (0, 0)	Median D = 0 (0, 0)	0.879
Percentage of REM sleep (%)^f^	0.0 (0.0, 0.1)	0.0 (0.0, 0.1)	Median D = 0 (0, 0)	0.872
Presence of REM sleep	7 (20.0%)	6 (18.2%)	RR = 0.98 (0.78, 1.23)	0.849
Arousal index (times/h)^g^	3.6 (2.5, 7.3)	2.7 (1.9, 4.9)	Median D = −0.9 (−2.2, 0.1)	0.091

Data are median (interquartile range) or n (%).

D, difference; N, non-rapid eye movement; REM, rapid eye movement; RR, relative risk.

a Sum of time spent in any sleep stage during the monitoring period.

b Calculated as the sum of each sleep stage divided by total sleep monitoring time.

c Calculated as the duration of N1 sleep divided by total sleep time.

d Calculated as the duration of N2 sleep divided by total sleep time.

e Calculated as the duration of N3 sleep divided by total sleep time.

f Calculated as the duration of REM sleep divided by total sleep time.

g Calculated as arousal times divided by total sleep time.

There were no significant differences between the two groups regarding the incidences of adverse events and the administered treatments, but slightly more patients in the dexmedetomidine group developed bradycardia [0.0% (0/40) with placebo vs. 10.0% (4/40); *P* = 0.110]. No severe adverse events occurred in both groups during the study period (Table 5).

**Table 5:** Safety outcomes.

	**Placebo group** **(*N* = 40)**	**Dexmedetomidine group** **(*N* = 40)**	***P*-value**
Bradycardia^a^	0 (0.0%)	4 (10.0%)	0.110
Premature interruption	0 (0.0%)	2 (5.0%)	0.494
Hypotension^b^	2 (5.0%)	1 (2.5%)	>0.999
Premature interruption	2 (5.0%)	0 (0.0%)	0.494
Tachycardia^c^	0 (0.0%)	0 (0.0%)	–
Hypertension^d^	0 (0.0%)	0 (0.0%)	–
Respiratory depression^e^	0 (0.0%)	0 (0.0%)	–
Desaturation^f^	0 (0.0%)	0 (0.0%)	–
Over sedation^g^	0 (0.0%)	0 (0.0%)	–

Data are n (%).

a Heart rate <50 beats per minute.

b Systolic blood pressure <90 mmHg or a decrease of more than 30% from baseline.

c Heart rate >120 beats per minute.

d Systolic blood pressure >180 mmHg or an increase of more than 30% from baseline.

e Respiratory rate <10 breaths per minute.

f Pulse oxygen saturation <90%.

g A score of Richmond Agitation-Sedation Scale ≤ -3; the score of Richmond Agitation-Sedation Scale ranges from −5 (unarousable) to +4 (combative), with 0 indicating an alert and calm subject.

## Discussion

Results of this pilot trial showed that for patients admitted to the ICU with invasive mechanical ventilation after major surgery, low-dose dexmedetomidine infusion did not significantly improve the subjective sleep quality and sleep architecture during the night of surgery, but there were trends of sleep improvement. Current results support the conduct of a large randomized trial.

Essential measures should be undertaken to improve the sleep quality in ICU patients, especially those following major surgery (16, 39). Non-pharmacological interventions are the first-line choice for this purpose and include improving the environment, reducing interruption from nighttime care, and using an appropriate mode of ventilation (16). As part of a multimodal approach, pharmacotherapy is often necessary to improve sleep. However, there are no recommended medications for sleep promotion in these patients until now (16). Although benzodiazepines and analgesics produce sedative effects, they result in an abnormal sleep architecture (40). There is no sufficient evidence that propofol or melatonin improves sleep quality (16). Previous studies suggest that sedative-dose dexmedetomidine infusion may be helpful to improve sleep of patients receiving invasive mechanical ventilation (41). However, frequent adverse events impede the widespread use of sedative dexmedetomidine in this patient population (20, 21). Indeed, in a recent trial of mechanically ventilated ICU patients, dexmedetomidine sedation was associated with significantly more adverse and even serious adverse events, of which the majority were bradycardia and hypotension (42).

In our previous studies, low-dose dexmedetomidine infusion at night improved sleep quality without increasing adverse events in postoperative ICU patients, the majority of whom did not receive invasive mechanical ventilation (22, 23). Subsequent studies showed that low-dose dexmedetomidine combined with opioids also improves analgesia and subjective sleep quality (43, 44). Chen et al. (45) reported that dexmedetomidine administered at a rate of 0.05–0.08 μg/kg/h *via* a patient-controlled analgesia pump improved the sleep structure (45). We thus tested the hypothesis that low-dose dexmedetomidine infusion might improve sleep quality in mechanically ventilated ICU patients after surgery. Our results showed that there was a trend toward an improved sleep quality pattern in patients of the dexmedetomidine group, which was characterized by slightly prolonged total sleep time, decreased stage N1 sleep and arousal index, and increased sleep efficiency and stage N3 sleep, as well as slightly improved overall RCSQ score. However, the differences were not statistically significant due to underpowered sample size. Daily results showed that dexmedetomidine only improved two RCSQ items and the overall RCSQ score on postoperative day 6. The effects of low-dose dexmedetomidine in improving sleep quality of patients with invasive ventilation are therefore promising but require further confirmation.

According to early experimental studies, dexmedetomidine exerts its sedative effects by activating the endogenous sleep-promoting pathway and produces a state like non-rapid eye movement sleep (17). A study in healthy volunteers also showed that dexmedetomidine induced electroencephalographic activities closely approximate to the process of sleep beginning, that is, increased slow-delta oscillations but decreased beta oscillations across the entire brain, as well as increased theta and spindle oscillations in occipital and frontal regions, respectively (46). Slow delta oscillations are characteristics of stage N3 sleep, spindle oscillations occur during stage N2 sleep, theta oscillations are characteristics of the later stage of N1 sleep, and beta oscillations appear in awakening (47, 48). In our results, the trends in the improvement of subjective sleep quality were in line with those of the objective sleep structure.

In the present study, low-dose dexmedetomidine infusion was continued during invasive mechanical ventilation in patients of the intervention group. Thus, although it may improve sleep quality at night, it may also lower the enthusiasm of critically ill patients to complete tasks, which should be monitored in future studies (49). Furthermore, the daytime cognitive functions, such as memory and attention, of critically ill patients should also be monitored in future studies in order to confirm the effects of night sleep promotion (50, 51). Nighttime administration might be a better strategy for sleep promotion even in patients with invasive ventilation. Recently, two trials showed that nocturnal dexmedetomidine infusion, rather than continuous administration, decreased delirium of critical ill patients during ICU stay (52, 53).

Among our patients, the incidence of adverse events did not differ between the two groups. This was in line with other studies investigating the effects of low-dose dexmedetomidine in ICU patients (22, 23, 51). We note that four patients of the dexmedetomidine group developed bradycardia (heart rate <50 beats per minute); of these, two required premature study interruption. Therefore, bradycardia might be a concern even when using low-dose dexmedetomidine in ICU patients.

There are some limitations to this pilot trial. First, as a pilot trial, the sample size was small. A larger sample study is needed to further evaluate the effect of low-dose dexmedetomidine infusion on sleep quality in this patient population. Second, 12 (15%) patients were excluded from sleep structure analysis due to failed polysomnographic monitoring; this may produce bias in our sleep structure results. However, dropouts were due to technical reasons, and it is difficult to avoid sleep monitoring failure in such studies.

## Conclusion

According to the results of this pilot trial, low-dose dexmedetomidine infusion did not significantly improve the sleep quality pattern in patients admitted to the ICU after surgery with intubation and mechanical ventilation, but there were trends of improvement. A large randomized trial is warranted to confirm the effects of low-dose dexmedetomidine in this patient population.

## Data availability statement

The raw data supporting the conclusions of this article will be made available by the authors, without undue reservation.

## Ethics statement

The studies involving human participants were reviewed and approved by the Clinical Research Ethics Committee of Peking University First Hospital [2017(13)]. The patients/participants provided their written informed consent to participate in this study.

## Author contributions

Y-MS, S-LL, and D-XW: research idea, study design, data analysis, and interpretation. Y-MS: data acquisition and manuscript drafting. Y-MS and S-NZ: statistical analysis. S-LL and D-XW: supervision or mentorship and critical revision. All authors contributed to the manuscript for important intellectual content and approved the final version.

## Funding

This work was supported by China Health Information and Health Care Big Data Association Severe Infection Analgesia and Sedation Big Data Special Fund No. Z-2019-1-004 (Beijing, China) and Cross Clinical Research Foundation of Peking University First Hospital No. 2017CR28 (Beijing, China).

## Conflict of interest

The authors declare that the research was conducted in the absence of any commercial or financial relationships that could be construed as a potential conflict of interest.

## Publisher's note

All claims expressed in this article are solely those of the authors and do not necessarily represent those of their affiliated organizations, or those of the publisher, the editors and the reviewers. Any product that may be evaluated in this article, or claim that may be made by its manufacturer, is not guaranteed or endorsed by the publisher.

## Abbreviations

BPS: behavior pain scale
CAM-ICU: Confusion Assessment Method for the ICU
DEX: dexmedetomidine
ECG: electroencephalogram
EEG: electroencephalograph
EMG: three-channel chin electromyogram
EOG: two-channel electrooculogram
NREM: non-rapid eye movement
NRS: numeric rating scales
OSAS: obstructive sleep apnea syndrome
PSG: polysomnography
PSQI: Pittsburgh Sleep Quality Index
RASS: Richmond Agitation-Sedation Scale
RCSQ: Richards–Campbell Sleep Questionnaire
REM: rapid eye movement
TICS-m: Telephone Interview for Cognitive Status-modified
VAS: visual analog scale
WhoQOL-BREF: World Health Organization Quality of Life-brief version.
