# Supplementary material for: Effect of low-dose dexmedetomidine on sleep quality in postoperative patients with mechanical ventilation in the intensive care unit: A pilot randomized trial
Source: Front Med (Lausanne). 2022 Aug 31;9:931084. doi: 10.3389/fmed.2022.931084 (PMC9471089; doi:10.3389/fmed.2022.931084)
Supplement: Supplementary file 1 [file Table_1.docx]

**Supplement Table. Postoperative results of individual Richards-Campbell Sleep Questionnaire items ^a^**

|  | **Placebo Group (N=40)** | **P value ^b^** | **Dexmedetomidine Group (N=40)** | **P value ^b^** | **MD (95% CI) ^c^** | **P value ^d^** |
| --- | --- | --- | --- | --- | --- | --- |
| Sleep depth |  | 0.087 |  | 0.906 |  | 0.152 |
| Day 1 | 12 (8, 20) |  | 14 (8, 78) |  | 2 (-3, 11) |  |
| Day 2 | 14 (8, 81) |  | 15 (8, 74) |  | 0 (-6, 6) |  |
| Day 3 | 18 (12, 50) |  | 17 (10, 73) |  | 0 (-7, 7) |  |
| Day 4 | 21 (11, 82) |  | 12 (7, 56) |  | -6 (-12, 0) |  |
| Day 5 | 17 (10, 50) |  | 17 (11, 83) |  | 1 (-5, 6) |  |
| Day 6 | 13 (7, 23) |  | 18 (8, 81) |  | 5 (-1, 13) |  |
| Day 7 | 14 (7, 31) |  | 12 (9, 59) |  | 1 (-5, 5) |  |
| Sleep latency |  | 0.549 |  | 0.599 |  | 0.240 |
| Day 1 | 74 (9, 85) |  | 45 (12, 87) |  | 2 (-5, 6) |  |
| Day 2 | 74 (12, 83) |  | 71 (13, 87) |  | 2 (-6, 10) |  |
| Day 3 | 69 (14, 83) |  | 76 (15, 86) |  | 2 (-6, 11) |  |
| Day 4 | 34 (12, 82) |  | 50 (8, 86) |  | 0 (-9, 12) |  |
| Day 5 | 22 (8, 79) |  | 71 (15, 88) |  | 9 (-1, 27) |  |
| Day 6 | 32 (13, 84) |  | 70 (22, 91) |  | 9 (0, 27) |  |
| Day 7 | 72 (9, 87) |  | 72 (11, 91) |  | 3 (-5, 12) |  |
| Awakenings |  | 0.148 |  | 0.170 |  | 0.454 |
| Day 1 | 24 (12, 65) |  | 34 (15, 73) |  | 4 (-6, 15) |  |
| Day 2 | 47 (15, 80) |  | 30 (21, 65) |  | -3 (-19, 10) |  |
| Day 3 | 43 (12, 62) |  | 56 (17, 80) |  | 7 (-6, 24) |  |
| Day 4 | 41 (14, 63) |  | 32 (16, 70) |  | -3 (-15, 11) |  |
| Day 5 | 37 (14, 78) |  | 55 (21, 83) |  | 6 (-6, 20) |  |
| Day 6 | 43 (11, 73) |  | 51 (20, 88) |  | 9 (-4, 26) |  |
| Day 7 | 34 (16, 75) |  | 51 (17, 91) |  | 6 (-7, 21) |  |
| Returning to sleep |  | 0.547 |  | 0.900 |  | 0.929 |
| Day 1 | 23 (11, 80) |  | 69 (13, 86) |  | 5 (-3, 16) |  |
| Day 2 | 57 (8, 84) |  | 71 (19, 85) |  | 3 (-5, 15) |  |
| Day 3 | 44 (9, 79) |  | 67 (17, 87) |  | 6 (-5, 17) |  |
| Day 4 | 23 (13, 79) |  | 41 (13, 80) |  | 3 (-8, 16) |  |
| Day 5 | 18 (11, 67) |  | 31 (14, 82) |  | 7 (-3, 20) |  |
| Day 6 | 17 (8, 60) |  | 54 (17, 86) |  | 15 (3, 35) |  |
| Day 7 | 24 (10, 82) |  | 58 (12, 88) |  | 4 (-5, 14) |  |
| Overall sleep quality |  | 0.844 |  | 0.368 |  | 0.686 |
| Day 1 | 42 (12, 80) |  | 57 (15, 85) |  | 4 (-5, 15) |  |
| Day 2 | 60 (8, 78) |  | 48 (13, 84) |  | 5 (-8, 15) |  |
| Day 3 | 51 (10, 75) |  | 55 (11, 82) |  | 3 (-8, 16) |  |
| Day 4 | 42 (14, 81) |  | 40 (10, 79) |  | -2 (-14, 11) |  |
| Day 5 | 18 (13, 78) |  | 37 (22, 80) |  | 9 (-4, 20) |  |
| Day 6 | 28 (11, 77) |  | 72 (18, 88) |  | 13 (2, 36) |  |
| Day 7 | 31 (13, 83) |  | 74 (19, 90) |  | 7 (-3, 23) |  |
| Overall score |  | 0.606 |  | 0.792 |  | 0.526 |
| Day 1 | 45 (13, 65) |  | 49 (14, 72) |  | 5 (-5, 18) |  |
| Day 2 | 52 (17, 71) |  | 49 (21, 71) |  | 1 (-13, 13) |  |
| Day 3 | 51 (14, 66) |  | 53 (17, 76) |  | 4 (-8, 18) |  |
| Day 4 | 33 (15, 70) |  | 45 (12, 67) |  | -2 (-14, 12) |  |
| Day 5 | 26 (12, 67) |  | 38 (21, 68) |  | 9 (-4, 21) |  |
| Day 6 | 31 (15, 59) |  | 53 (22, 74) |  | 14 (1, 29) |  |
| Day 7 | 47 (12, 69) |  | 47 (12, 72) |  | 3 (-9, 19) |  |
| Noise |  | 0.564 |  | 0.270 |  | 0.646 |
| Day 1 | 82 (56, 86) |  | 83 (73, 90) |  | 3 (-2, 8) |  |
| Day 2 | 83 (66, 87) |  | 83 (76, 88) |  | 2 (-3, 7) |  |
| Day 3 | 79 (71, 87) |  | 84 (73, 90) |  | 2 (-3, 7) |  |
| Day 4 | 82 (76, 89) |  | 83 (69, 87) |  | 0 (-5, 5) |  |
| Day 5 | 84 (68, 89) |  | 82 (73, 89) |  | 0 (-5, 4) |  |
| Day 6 | 81 (77, 87) |  | 82 (72, 87) |  | 1 (-4, 5) |  |
| Day 7 | 80 (68, 87) |  | 84 (79, 91) |  | 5 (0, 11) |  |

Data are median (interquartile range). MD, median difference.

^a^ Richards-Campbell Sleep Questionnaire is a 5-item questionnaire. Responses are recorded on a 100-millimeter visual-analogue scale, with higher scores representing better sleep and the mean of these five items representing the overall score (primary measure). The Richards-Campbell Sleep Questionnaire also included a sixth item, not included in the overall score, evaluating perceived nighttime noise (visual-analogue scale range: 0 for “very noisy” to 100 for “very quiet”).

^b^ For the effect of time within group.

^c^ Calculated as dexmedetomidine group minus placebo group.

^d^ For the effect of group × time.

**Contribution to the field**

1. This pilot trial tested the effect of low-dose dexmedetomidine infusion on night-time sleep quality in mechanically ventilated ICU patients after surgery.

2. The results showed that, among patients admitted to the ICU after surgery requiring mechanical ventilation, low-dose dexmedetomidine infusion did not significantly improve sleep quality pattern but there were trends of improvement.

3. Findings from this pilot trial support the conduct of a large randomized trial to investigate the effect of low-dose dexmedetomidine in this patient population.
